# λ-Carrageenan Suppresses Tomato Chlorotic Dwarf Viroid (TCDVd) Replication and Symptom Expression in Tomatoes

**DOI:** 10.3390/md13052875

**Published:** 2015-05-08

**Authors:** Jatinder S. Sangha, Saveetha Kandasamy, Wajahatullah Khan, Navratan Singh Bahia, Rudra P. Singh, Alan T. Critchley, Balakrishnan Prithiviraj

**Affiliations:** 1 Department of Environmental Sciences, Faculty of Agriculture, Dalhousie University, P.O. Box 550, Truro, NS B2N 5E3, Canada; E-Mails: Jatinder.sangha@dal.ca (J.S.S.); skandasamy@dal.ca (S.K.); pulsarsidhu@gmail.com (N.S.B.); 2 Basic Sciences Department, King Saud Bin Abdul Aziz University for Health Sciences, P.O. Box 22490, Riyadh 11426, Saudi Arabia; E-Mail: Khanmuh@ksau-hs.edu.sa; 3 Agriculture and Agri-Food Canada, 850 Lincoln Rd., Fredericton, NB E3B 4Z7, Canada; E-Mail: singhr@agr.gc.ca; 4 Acadian Seaplants Limited, 30 Brown Avenue, Dartmouth, NS B3B 1X8, Canada; E-Mail: alan.critchley@acadian.ca

**Keywords:** tomato chlorotic dwarf viroid (TCDVd), carrageenans, induced resistance

## Abstract

The effect of carrageenans on tomato chlorotic dwarf viroid (TCDVd) replication and symptom expression was studied. Three-week-old tomato plants were spray-treated with iota(ɩ)-, lambda(λ)-, and kappa(κ)-carrageenan at 1 g·L^−1^ and inoculated with TCDVd after 48 h. The λ-carrageenan significantly suppressed viroid symptom expression after eight weeks of inoculation, only 28% plants showed distinctive bunchy-top symptoms as compared to the 82% in the control group. Viroid concentration was reduced in the infected shoot cuttings incubated in λ-carrageenan amended growth medium. Proteome analysis revealed that 16 tomato proteins were differentially expressed in the λ-carrageenan treated plants. Jasmonic acid related genes, allene oxide synthase (AOS) and lipoxygenase (LOX), were up-regulated in λ-carrageenan treatment during viroid infection. Taken together, our results suggest that λ-carrageenan induced tomato defense against TCDVd, which was partly jasmonic acid(JA) dependent, and that it could be explored in plant protection against viroid infection.

## 1. Introduction

Viroids are the smallest (246–400 nucleotides) single-stranded, non-protein-coding circular RNA molecules [1]. Since their initial discovery in 1971 [2,3], almost 30 types of viroids belonging to the Pospiviroidae and Avsunviroidae families have been reported to infect a wide array of plants, and the number may increase with the discovery of additional hosts [4]. The genus *Pospiviroid*, in the family Pospiviroidae, contains about nine viroid species, including tomato chlorotic dwarf viroid (TCDVd) [1], which causes more than 25 diseases in agricultural, horticultural and ornamental plants [5]. Due to their destructive nature, there is growing interest to develop strategies to control viroids. Although considerable advances have been made in the characterization of viroids, mechanisms of viroid pathogenicity and symptom expression [6,7,8,9], mechanisms of plant resistance to viroid infection is not understood.

Unlike plant resistance to most other pathogens, natural resistance against viroids is not common [10]. Attempts have been made to protect plant against viroids using different strategies: non-transgenicapproaches such as detection and eradication of viroid-infected plants [11], chemical-induced resistance [12,13], cross protection [14], thermotherapy [15,16], and tissue culture and grafting [15,16,17] showed variable levels of success in controlling viroid diseases. Crop germplasms have been screened, and resistant clones and cultivars that showed tolerance to viroids have been reported [11,18,19]. Plant breeding techniques offered promising results in some cases such as chrysanthemum stunt viroid (CSVd) resistance in chrysanthemums [20,21], although it may not be possible in other crop species due to lack of resistant germplasms. Recent techniques in molecular biology, such as RNA silencing, showed a reduction in the replication of viroids [17,22], thus suggesting the potential use of this technique in developing viroid resistant varieties through genetic engineering.

Plants deploy a wide range of defense mechanisms against pathogens. These defense mechanisms can be either constitutive or inducible that prevents pathogen ingress [23]. Upon perceiving pathogen attack, usually by the recognition of pathogen-specific elicitors, plants activate defense responses against the pathogen [24]. This may involve a series of molecular events in the pathogen-challenged plants; regulation of the production of plant hormones, secretion of defensive enzymes or accumulation of PR proteins in plant cells [24,25,26]. Plant defense responses can also be induced by cell wall components of pathogen like chitin, lipo-polysaccharides, bacterial flagellin proteins and other chemicals of natural and synthetic origin like salicylic acid, jasmonic acid, isonicotinic acid and benzothiadiazole [27,28,29]. Compounds that induce plant defense responses, referred to as elicitors, have been identified in a number of seaweeds. Examples of such compounds include laminarin, fucans, ulvans and carrageenans [28,30,31].

Carrageenans are sulfated polysaccharides that are the major cell wall component of red seaweeds [32]. In some red seaweeds, carrageenans may account for more than 40% by dry weight. Carrageenans are grouped as iota(ι)-, kappa(κ)- and lambda(λ)-carrageenan based on the degree of sulfation, and each one presents its own characteristic bioactivity [31]. An emerging body of literature suggests that carrageenans inhibit binding of viruses like human papillomavirus (HPV) to the animal cells, which is attributed to its structural similarity to heparan sulfate, an HPV cell-attachment factor [33]. Carrageenans have been shown to elicit resistance in plants and animals against pathogens, and this activity depends on the degree of sulfation [28,30,34,35,36]. In this study, we tested the effect of three types of carrageenans (ι, κ, and λ) on the induction of resistance in tomatoes against TCDVd.

## 2. Results

### 2.1. Effect of Carrageenans on TCDVd Infection in Tomatoes

**Figure 1:**
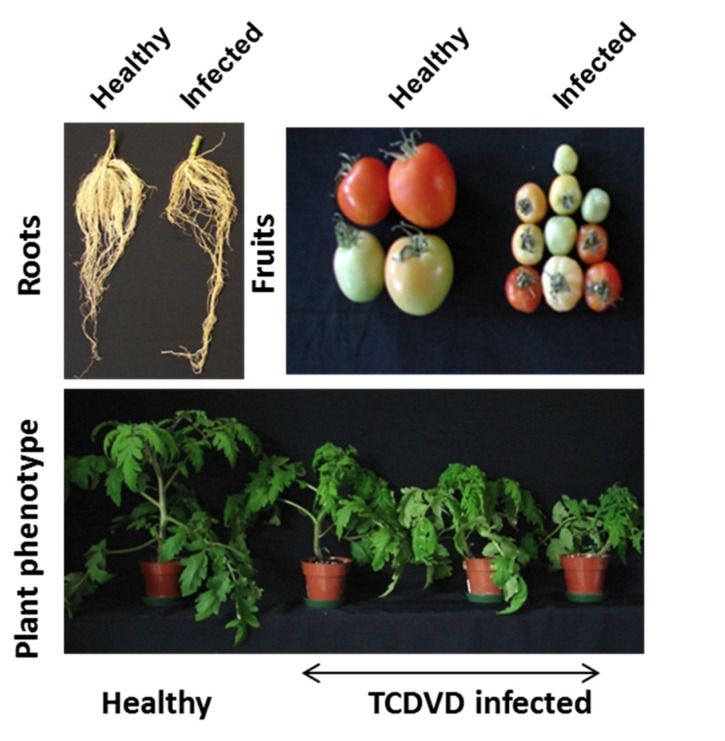
Symptoms of TCDVd infection in tomatoes. Three-week-old tomato plants were inoculated with 10 μL of TCDVd sap and the symptoms were observed at 28 dpi. Infected plants (RT-PCR confirmed) were stunted with bunchy top symptoms, smaller fruits and fewer roots.

Stunting and bunchy tops are the most characteristic symptoms induced by TCDVd infection in tomatoes. The infected plants showed variable phenotypic symptoms and were positive for TCDVd as confirmed with reverse transcription polymerase chain reaction (RT-PCR). The fruit size was also considerably reduced and roots were thin and fibrous in the infected plants (Figure 1).

**Figure 2:**
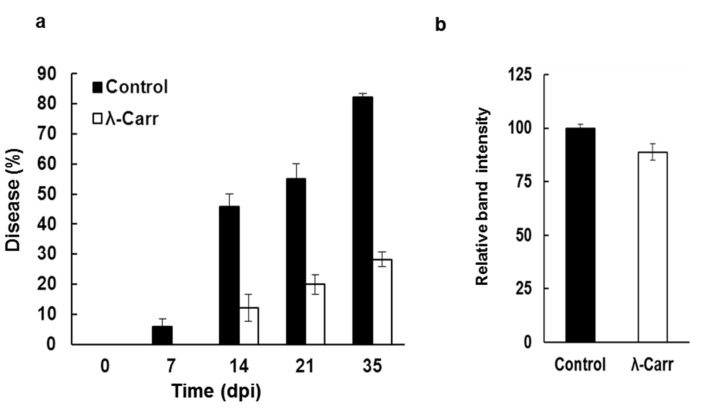
Suppressive effect of λ-carrageenan in tomatoes against TCDVd infection. (**a**) Percentage of plants showing typical TCDVd symptoms in control and λ-carrageenan (λ-Carr) treated plants. Data represent the mean of percent infected plants from three independent trials (Mean ± SEM, *n* = 36); (**b**) Relative intensity of TCDVd bands visualized on agarose gel and quantified with ImageJ software in controls and λ-carrageenan (λ-Carr) treated plants at 35 dpi (Mean ± SEM, *n* = 10).

To determine if carrageenans reduced TCDVd infection or prevented symptom development in tomatoes, three-week-old tomatoes (cv. Sheyenne) plants were treated with carrageenans (ɩ, κ, λ) and then inoculated with TCDVd. No difference in viroid replication or symptom development was observed in ɩ- and κ-carrageenan treated plants. The λ-carrageenan treatment, however, protected the tomatoes from TCDVd infection (Figure 2a) and decreased the replication of TCDVd in the plant tissue as observed with RT-PCR. Based on the phenotype of the plant at 35-days post inoculation (dpi), a higher number (82%) of untreated plants were infected with TCDVd, whereas less than 30% plants that were treated with λ-carrageenan exhibited TCDVd symptoms. Further analysis was performed with RT-PCR using TCDVd specific primers on plants at 0, 14 and 35 dpi. The RT-PCR analysis detected TCDVd transcripts in plants which otherwise appeared normal, particularly at an early stage, *i.e.*, one to two weeks after inoculation. The results indicated that the viroid infection was established in untreated plants by 14 dpi as 46% of plants showed TCDVd transcript at this stage. The number of infected plants in the untreated group increased to 55% at 21 dpi and to 82% at 35 dpi (Figure 2a). In contrast, TCDVd infection was delayed or suppressed in λ-carrageenan treated plants; none of the plants showed TCDVd symptoms at 7 dpi; further, there were no viroid transcripts in the leaves. Fewer plants (12%) developed TCDVd symptoms at 14 dpi and the number of infected plants increased to 20% at 21 dpi, and 28% at 35 dpi. Interestingly, fewer plants were visibly stunted in λ-carrageenan treatment at 35 dpi even though the plants had TCDVd transcripts, suggesting that λ-carrageenan suppressed symptom expression.

To confirm whether the reduction in the severity of symptoms in λ-carrageenan treated plants was associated with the reduction in viroid multiplication, the abundance of TCDVd transcripts was measured using RT-PCR in leaf samples collected at 35 dpi (Figure 2b). The intensity of TCDVd specific bands in the treated plants was quantified using ImageJ software (National Institutes of Health, MD, USA). The result revealed that the average (*n* = 10) band intensity of TCDVd in control plants was higher than in the λ-carrageenan treated plants which conformed with the reduced symptoms in the λ-carrageenan treated plants. These results suggest suppressive effects of λ-carrageenan on TCDVd in-planta.

### 2.2. Effect of Carrageenan Treatments on Plant Height

**Figure 3:**
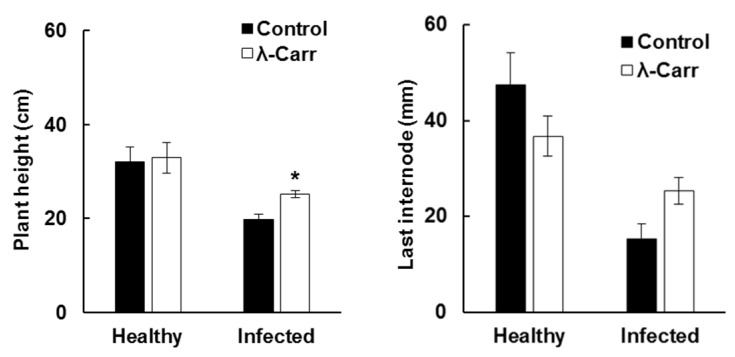
Plant height and last internode length of infected and healthy tomato plants one month after TCDVd inoculation. Bars represent control (black) and λ-carrageenan (λ-Carr, white). Data represent Mean ± SEM (*n* = 36 for healthy plants and *n* = 12 plants for infected plants), student’s *t*-test at *p* < 0.05.

Viroid infection had a negative effect on the growth and development of plants causing reduced plant height (stunting), short internodes, initiation of new shoots and reduced fruit size. We measured the height of TCDVd-inoculated plants to determine the protective effect of λ-carrageenan against viroid infection. Although the average height of TCDVd infected plants, after one month of infection, was reduced in all treatments compared to non-inoculated plants, the height of λ-carrageenan treated plants was significantly higher (*p* < 0.05) (Figure 3). The average height of TCDVd infected plants treated with λ-carrageenan was ~25 cm compared to ~20 cm in the control. Since viroid infection reduced internode length, we compared the length of the last internode in λ-carrageenan treated and control plants, although the internode length was higher in λ-carrageenan treated plants, the difference was not significant (Figure 3).

### 2.3. Effect of λ-Carrageenan on TCDVd Replication

**Figure 4:**
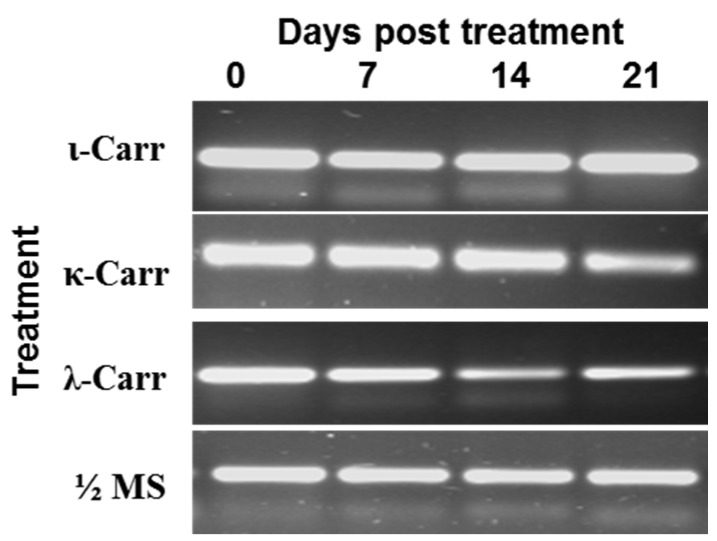
Effect of carrageenans on TCDVd multiplication in tomato determined by RT-PCR. TCDVd concentration in the leaf samples from infected shoots was determined at 0, 7, 14, and 21 days post carrageenan treatment (λ-carrageenan (λ-Carr), ɩ-carrageenan (ɩ-Carr), κ-carrageenan (κ-Carr)) with RT-PCR using TCDVd specific primers. The bands were visualized on agarose gel.

To verify if λ-carrageenan had a remedial effect on TCDVd-infected plants that could inhibit the replication of TCDVd, shoot cuttings from TCDVd-infected plants were incubated in the λ-carrageenan amended plant growth medium (½ strength Murashige & Skoog (MS) medium) for three weeks and the TCDVd concentration in the shoot was determined at weekly intervals by RT-PCR. The viroid concentration in the leaves of the infected shoot gradually reduced in the plant shoot incubated in the λ-carrageenan solution (Figure 4). However, other carageenans, *i.e.*, ɩ- and κ-carrageenan, did not reduce the concentration of TCDVd concentration in the shoots. Interestingly, ɩ-carrageenan increased the viroid concentration in the shoot (Figure 4).

#### 2.3.1. λ-Carrageenan Induced Differential Expression of Tomato Proteins

**Figure 5:**
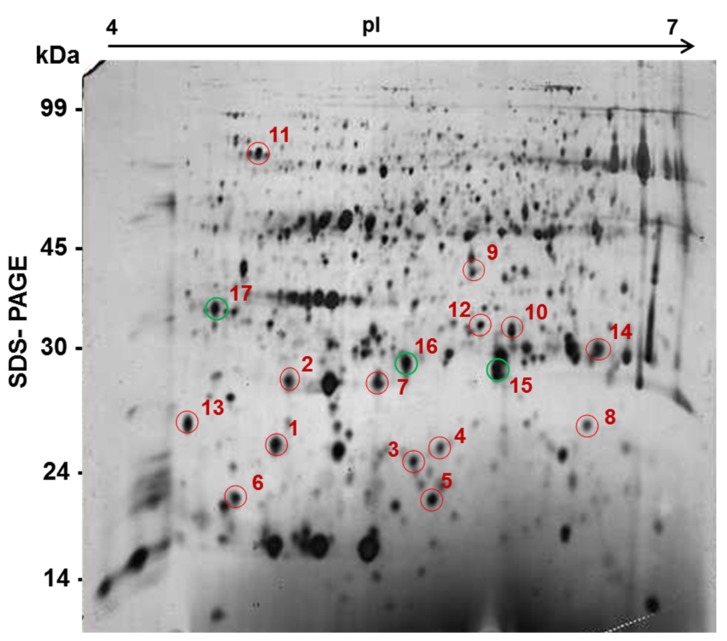
A general proteome map of tomato leaves with TCDVd infection in λ-carrageenan treatment. Proteins were separated first on an IPG strip (pH 4.0–7.0) and then based on molecular weight (kDA) on a 12% SDS-PAGE. Differentially expressed proteins are marked, red circled were increased whereas green circled were decreased.

Plant defense response to TCDVd infection was analyzed by a proteomics approach using 2D-gel electrophoresis. The proteome of the leaf tissue of control and λ-carrageenan treated plants inoculated with TCDVd were analyzed. Compared to untreated (control) plants, λ-carrageenan elicited plants showed differential expression of proteins in response to TCDVd infection (Figure 5, Table 1). Seventeen proteins were differentially expressed in control and λ-carrageenan treated tomato plants inoculated with TCDVd. The results showed that 14 proteins increased in abundance in λ-carrageenan treated plant whereas three proteins were reduced. These proteins have different functions, suggesting that TCDVd resistance in tomatoes involved multiple pathways to defend against the infection.

#### 2.3.2. Effect of λ-Carrageenan on the Expression of Defense Response Genes during Viroid Infection

**Figure 6:**
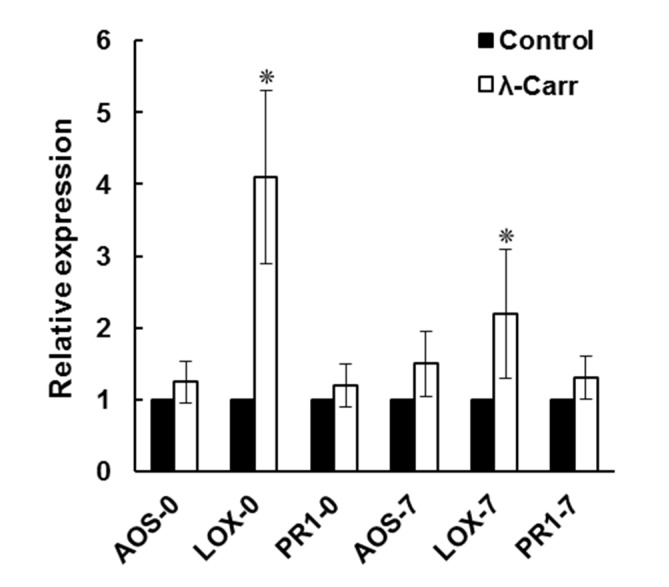
Expression of defense response genes encoding allene oxide synthase (AOS), lipoxygenase (LOX) and pathogenesis related protein 1 (PR1), in tomatoes during TCDVd infection. The expression of genes was analyzed at 0 (AOS-0, LOX-0 and PR1-0) and 7 (AOS-7, LOX-7 and PR1-7) dpi in control and λ-carrageenan (λ-Carr) treated plants. Values with “*” are significantly different (*p* < 0.05). (Mean ± S.E., *n* = 3).

Plant defense genes are involved in providing protection during challenge by various biotic stresses including pathogens. Some of these genes may be involved in carrageenan-induced defense in tomatoes. We examined the expression of three defense genes (*TomLoxD, TomAOS* and *PR1*) in control and λ-carrageenan-treated tomato plants at different time points after TCDVd inoculation (Figure 6). The results revealed that the λ-carrageenan-elicited defense response against TCDVd is associated with JA-dependent plant defense genes as both *TomAOS* and *TomLoxD* being up-regulated in λ-carrageenan treatment in TCDVd infected plants at 0 and 7 dpi, respectively. In contrast, *PR1* response was not different in λ-carrageenan treated tomato plants as compared to the control group.

**Table 1:** List of leaf proteins differentially expressed in λ-carrageenan treated tomato with tomato chlorotic dwarf viroid (TCDVd) infection as compared to control plants with TCDVd infection (*p* < 0.05).

Spot ID	Protein Identity	Accession	Molecular Mass (kDa)	Isoelectric Point (pI)	Fold Change	No. of Unique Peptides	Coverage (%)
Proteins over expressed in λ-carrageenan treated tomato with viroid infection							
1	Cytochrome b6-f complex iron-sulfur subunit	Q69GY7-UCRIA_SOLTU	24.2	4.85	2.39	3	12.6
2	Photo-system II oxygen-evolving complex protein 2	Q7M1Y7-Q7M1Y7_ORYSA	04.0	4.90	2.37	1	35.1
3	Pathogenesis-related protein 10	Q4KYL1_ORYSA	17.5	5.59	2.45	3	6.82
4	17.7 kDa class I small heat-shock protein	A1E463_9ASTR	17.5	5.73	1.92	1	14.3
5	Superoxide dismutase 1	P14830-SODC1_SOLLC	15.2	5.66	2.70	5	23.7
6	17.7 kDa classI heat shock protein	O82011-HSP11_SOLPE	17.7	4.65	2.43	3	18.8
7	Ribulose bisphosphate (RuBP) carboxylase small chain Fragment	A0A3A2_ARTAN	19.7	5.40	2.35	1	4.62
8	Superoxide dismutase	Q6X1D0_SOLLC	27.9	6.45	2.90	4	15.3
9	Cytosolic cysteine synthase	Q9FS27_SOLTU	34.2	5.92	1.99	2	7.38
10	Proteasome subunit alpha type	Q93X34_TOBAC	27.1	6.21	2.49	5	18.5
11	Uracil phosphoribosyltransferase	P93394-UPP_TOBAC	24.1	4.73	2.60	3	16.1
12	Cytosolic ascorbate peroxidase 1	B1Q3F7_SOLLC	27.3	6.00	2.85	9	37.6
13	Succinic semialdehyde reductase isoform 2	B1Q3F7_SOLLC	38.2	4.51	1.88	1	2.75
14	Ferredoxin-NADP reductase, leaf-type isozyme, chloroplastic	O04977-FENR1_TOBAC	40.4	6.62	1.73	3	23.8
Proteins under expressed in λ-carrageenan treated tomato with viroid infection							
15	Carbonic anhydrase	Q5NE20_SOLLC	34.4	6.21	1.79	3	10.6
16	Peptidyl-prolyl *cis*-*trans* isomerase	A0MTQ0_SOLSG	17.9	5.72	1.97	1	7.02
17	Germin like protein	B9A6I8_TOBAC	21.9	4.68	2.39	1	4.27

## 3. Discussion

Viroid RNA has been extensively studied due to their unique structure and infectious nature. However, there are only a few studies on the control of viroid diseases. This study demonstrates the potential use of a sulfated polysaccharide, λ-carrageenan, to induce resistance in tomatoes against TCDVd, a devastating pathogen on a number of plants [37]. The effect of λ-carrageenan appears to be plant-mediated as it did not show a direct effect on viroid RNA *in vitro*; gene expression analysis, and the analysis of the proteomes of the infected plants suggest that λ-carrageenan induced resistance against TCDVd was largely mediated by a jasmonic acid dependent defense pathway.

Use of elicitors to induce plants’ resistance against pathogens has been an effective strategy to reduce disease. Elicitors such as INA (2, 6-dichloro-isonicotinic acid), BTH (benzothiadiazole) and BABA (β-aminobutyric acid) induce transcription of specific defense genes [38,39]. Few previous reports suggested the effect of chemicals to protect plants against viroid infection. For example, the use of piperonyl butoxide showed protective effects against potato spindle tuber viroid infection [12]. Antiviral agent ribavirin (300 mg/l), when applied on *Gynura aurantiaca* (Purple Passion) plants infected with citrus exocortis viroid (CEVd), completely suppressed symptoms in newly developed leaves, whereas the application of the same agent three days prior to infection also prevented establishment of infection in the plant [13]. Pretreatment of tomato plants with λ-carrageenan reduced the TCDVd symptom expression, and the progression of the disease was also suppressed. Algal polysaccharides, such as laminarin and carrageenans trigger host defense mechanisms against subsequent pathogenic infections in plants and animals [31,33,36]. The application of κ/β-carrageenan from red marine alga *Tichocarpus crinitus* showed a significant reduction in the number of necrotic lesions on the tobacco leaves inoculated with the mixture of tobacco mosaic virus (TMV) [40]. In the present study, we observed that the highly sulfated λ-carrageenan induced tomato resistance against TCDVd suggesting the role of sulfation in the bioactivity as shown in earlier studies [28,31,41].

TCDVd virulence is dependent on the pathogenicity factors present in the viroid sequence [42]. We also investigated the TCDVd sequence isolated from a number of λ-carrageenan treated plants and did not find any mutational effect that could have caused reduced virulence of the viroid in tomato.

Proteome analysis revealed differential induction of proteins in TCDVd-infected λ-carrageenan treated plants. Several studies have revealed differential responses of plant proteomes in response to disease progression [43]. The proteome changes in plant show that the reduction in infection was primarily mediated by biochemical changes elicited by λ-carrageenan. In a previous study, Itaya *et al.*, [6] showed changes in host gene expression at different stages of the viroid infection and revealed a complex pattern of molecular changes in plant that involve defense responses, metabolic changes in cell wall and proteins and many other miscellaneous functions. Our proteomics results also revealed that tomato proteins involved in various physiological processes were altered indicating a complex nature of viroid-plant interactions. The higher expression of 14 proteins in λ-carrageenan treatment suggests their potential role in tomato defense to TCDVd that should be further investigated.

Results on the expression of lipoxygenase (*LOX*), allene oxide synthase (*AOS*) and pathogenesis-related protein (*PR1*) genes in TCDVd-infected tomatoes suggest a possible role of JA response in plants’ resistance against viroid infection. JA response has been important against various biotic stresses in plants. Wang *et al.* [7] investigated the expression of genes in a susceptible and a transgenic (resistant) tomato with potato spindle tuber viroid (PSTVd) infection and revealed several differentially expressed genes in tomatoes and a possible role of jasmonic acid biosynthesis pathway in tomato resistance to viroids. The induction of these two genes (*LOX* and *AOS*) by λ-carrageenan-treated tomatoes support the role of JA dependent signaling pathway in tomato defense against TCDVd. In addition, salicylic acid-inducible *PR1* in control was not different than λ-carrageenan treatment, indicating that jasmonic acid/ethylene is important in viroid-tomato interaction, a finding that needs further understanding.

## 4. Material and Methods

### 4.1. Plant and Viroid Culture

Tomato (*Solanum lycopersicum* cv. Sheyenne) seeds and tomato chlorotic dwarf viroid (TCDVd)-infected plants were obtained from the Potato Research Centre, Fredericton, Canada. The viroid culture was maintained on tomatoes (cv. Sheyenne) in a greenhouse at 24 ± 2 °C with a photoperiod of 16 h light and an 8 h dark cycle. For experiments, surface sterilized healthy tomato seeds were planted in 8′′ pots containing peat soil (Pro-mix) and maintained in the greenhouse under same conditions as described above.

### 4.2. Treatments

Three types of carrageenans (iota (ι), kappa (κ) and lambda (λ)) used in this study were provided by Cargill Texturant Solutions (Baupte, France). The carrageenans differed in sulfation, λ-carrageenan (35%) and κ*-*carrageenan (<30%). Spray solution were prepared by dissolving carrageenans in ultra-pure water (MilliQ) (0.1% w/v) containing 0.02% (v/v) of Tween-20.

### 4.3. Inoculation of Tomato Plant with TCDVd

Three-week-old tomato plants (8–10 cm tall) with fully expanded third leaves were sprayed with carrageenans until drip using a hand held atomizer. The control plants were treated in a similar manner with water containing Tween-20. Leaves (1 g) from TCDVd infected tomato were ground in 5 mL of 1:10 dilution (w/v) of buffer containing glycine (0.05 M) + dibasic potassium phosphate (0.03 M), pH 9.2 and the resulting sap was used for inoculating plants 48 h after spray treatment. Briefly, 10 μL each of TCDVd sap was rubbed gently over two leaflets of the second leaf for each treatment using a flame sterilized glass rod [37]. Inoculated plants were maintained in the growth chamber at 22 °C, 16 h:8 h for day:night conditions. Data on plant height and internode length were recorded at 21 dpi, whereas the appearance of bunchy top symptoms were observed on plants at 7, 14, 21, and 35 dpi [37].

### 4.4. Effect of Carrageenans on TCDVd in Infected Shoots

To investigate the effect of carrageenans on viroid multiplication, tomato shoot cuttings (5–6 cm long with 2 internodes) were excised from TCDVd infected plants, washed several times with distilled water and the cut end of the shoot was dipped in a 20 mL glass tube containing half strength Murashige & Skoog (MS) (Sigma, St. Louis, MO, USA) solution and incubated for two days at 24 ± 2 °C with a photoperiod of 16 h light and 8 h dark cycle. The MS medium was removed and replaced with MS solution supplemented with 1 g·L^−1^ carrageenans (ɩ, κ, λ). TCDVd concentration in the shoots was determined with RT-PCR (as described below) in 20 mm leaf disc excised from upper leaf at 0 (just before treatment), 1, 2, and 3 weeks post-treatment. Each treatment had three replications and the experiment was repeated twice.

### 4.5. Viroid Nucleic Acid Extraction and RT-PCR

The viroid RNA was extracted from the leaf tissue following published method [37]. Briefly, 100 mg of leaf tissue was homogenized in 500 μL viroid extraction buffer (50 mM NaOH + 2.5 mM EDTA) in a bead beater (Micro Smash MS-100, Tomy Co., Tokyo, Japan), set at 3000 rpm for 2 min. The homogenate was centrifuged 8000× *g* at 4 °C for 20 min and the supernatant (300 μL) was precipitated with 300 μL of isopropanol and 0.1 μL of 3 M sodium acetate (−20 °C overnight). The precipitate was collected by centrifugation (12,000× *g* at 4 °C) for 5 min, washed with 70% ethanol, air-dried, and dissolved in 150 μL of RNAase free water. Two μg RNA was reverse transcribed using High Capacity cDNA Reverse Transcription kit (Applied Biosystems, ON, Canada) with a Pospiviroid reverse primer (5′-AGCTTCAGTTGTTTCCACCGG GT-3′).

Polymerase chain reaction was carried with 2 μL of cDNA using Taq DNA polymerase (Applied Biosystems, Foster City, CA, USA) using the forward primer (5′-ATTAATCCCCGGGGAAACCTGGAG-3′) and reverse primer (5′-AGCTTCAGTTGTTTCCACCGGGT-3′) [44]. PCR was performed with standard conditions, except that 27 cycles were used to avoid saturation of the product. For quantification, ten microliters of amplified product was electrophoresed on 2% agarose gel containing 0.5 μg/mL ethidium bromide and photographed under UV light in a gel documentation system (Quantity One, Gel Doc EQ, Bio-Rad, Hercules, CA, USA). TCDVd band intensities were quantified by ImageJ free software (Version 1.33; http://rsb.info.nih.gov/ij/) and compared relative to the control.

### 4.6. Carrageenan Induced Defense Gene Expression in Tomatoes

To determine the response of tomato plant to carrageenan treatment following viroid inoculation, the transcript abundance of tomato defense genes *TomLOX* (forward primer, 5′-GGCCACGTTGACCTC CGCAA-3′; reverse primer, 5′-TGCGCTGAAGCCAGCCAGAT-3′, *TomAOS* (forward primer, 5′-ACACGACGCCGTTTTCGAGGTG-3′; reverse primer, 5′-CCGACGAACCGATCGGCGAC-3′ and *PR1* (forward primer, 5′-GACGAGTTGGCGTTGGCCCT-3′; reverse primer, 5′-AGCGGCTAGGTTTTCGCCGT-3') were analyzed by quantitative real time PCR. Leaf tissues were sampled at 0 and 7 dpi and total RNA was extracted using a plant RNA extraction kit (Qiagen, Mississauga, ON, Canada). The quality and quantity of RNA was assessed with Nanodrop ND-1000 (NanoDrop Technologies Wilmington, DE, USA) and formaldehyde gel electrophoresis. DNAase treated RNA was used to synthesize cDNA using Quantiscript reverse transcriptase kit (Qiagen, Mississauga, ON, Canada) following manufacturer’s instructions. Real-time PCR was performed on StepOne™ Real-Time PCR System (Applied Biosystems, Foster City, CA, USA) using SYBR green dye with Rox (Roche Diagnostics, Mississauga, ON, Canada) according to manufacturer instructions. Transcript abundance of each selected gene was normalized to18S ribosomal RNA. Data were analyzed from three independent Real-Time PCR runs.

### 4.7. Effect of Carrageenan on Tomato Proteomes

Leaf samples from tomato plants spray treated with carrageenans and inoculated with TCDVd as described above were harvested at 3 weeks after viroid inoculation, flash frozen in liquid nitrogen and ground to a fine powder. The total protein was extracted using a solution containing trichloroacetic acid (10%), acetone (89.93%) and dithiothreitol (0.07%). Protein concentration was determined by the Bradford Method [45] and stored in 100 μL aliquots at −80 °C until use.

For protein profiling, 100 μg of total proteins from the control and carrageenan-treated samples were separated with first dimension electrophoresis using 17 cm IPG strips, pH 4–7 at 500 V for 1 h, followed by 1000 V for 1 h and 1750 V for 24 h. The proteins were separated by SDS-PAGE in the second dimension using 12% polyacrylamide gels on a multiphor unit (Amersham Biosciences, Piscataway, NJ, USA). The gels were silver stained following published protocol [46]. The gels were scanned with a GS-700 imaging densitometer (Bio-Rad, Hercules, CA, USA) and analyzed with PD Quest software (Bio-Rad, Hercules, CA, USA).

For protein identification, differentially expressed protein spots were excised from the preparative gels and digested with trypsin using the MassPREP station (Waters, Milford, MA, USA). Protein identification and sequencing were carried out using two-dimensional liquid chromatography ESI MS (Agilent 1100 series 2D nano LC MS). The peptide mass data were subjected to the MASCOT search engine (Agilent, Santa Clara, CA, USA) for analysis. MS/MS spectra were used to search protein identity at NCBI non-redundant protein database using MS/MS Ion Search Engine at http://www.matrixscience.com/search_form_select.html. A single protein, having a higher score than the minimum score for the significance level (*p* < 0.05), was judged as a significant match.

### 4.8. Statistical Analysis

For data analysis, JMP-IN (Ver. 5) statistical software was used. Data were subjected to analysis of variance (ANOVA) and the means were compared for significance using Tukey’s test or student’s *t*-test (*p* < 0.05). Each experiment was replicated two to three times in independent trials. Values are represented as mean ± SEM (standard error of the mean).

## 5. Conclusions

In summary, the results showed that highly sulfated λ-carrageenan suppressed TCDVd in tomatoes by eliciting plant defense responses while less sulfated ɩ- or κ-carrageenan did not have an effect. The induction of plant resistance with λ-carrageenan could be a novel, cost effective and environmentally friendly approach for the management of this pathogen. Since pospiviroids are replicated in the nucleus by DNA-dependent RNA polymerase II, it remains unclear if λ-carrageenan affected this enzyme *in vivo*, which might have resulted in the reduced viroid concentration in the elicited plants.
